# Numerous Items

**Published:** 1896-09

**Authors:** 


### Medical News and Miscellany.

**The** *S. W. Medical Record* says, "Dr. Daniel is presump*tious*." Don't call us names, doctor; please.

**Lost.**—One of our Texas contemporaries has lost (or mislaid) his office dictionary. Any one finding it will please return to the *Northwestern Medical Record.* N. B. Good as new; *not much used.*

**Austin District Medical Society.**—The thirty-sixth quarterly meeting of the Austin District Medical Society will be held in Austin on Thursday, September 24th, 1896. An unusually interesting programme has been arranged, and the members of the regular profession are cordially invited to meet with us and participate in the discussions. We promise you a pleasant and profitable time.

S. E. HUDSON, Secretary.

**Prof. Lydston's** article (under head of Chicago Medico-Legal Society), is excellent reading. If disseminated as a tract, it would doubtless do much good.

---

**Dr. Ralph Steiner,** of Austin, Texas, United States Consul to Munich, we learn on reliable authority, will resign and return to Texas October 1, prox.

---

**A Sermon in Four Lines.**—"We have millions for foreign missions, millions for courts of law and penal institutions, but nothing for the salvation of those children of to-day who will be criminals of the future."—[Lydston, in this issue.]

---

**Dr. Love's Resignation.**—The faculty of the Marion-Sims Medical College passed the following resolution:

WHEREAS, Dr. I. N. Love has found it incumbent on himself to sever his connection with the Marion-Sims College of Medicine, the members of the faculty of that institution embrace this occasion to express their appreciation of his past services, and to extend to him their hope that in his future connections he will find both pleasure and profit.

S. M. HYPES,
R. C. ATKINSON,
C. BARCK,
Committee.

---

**Strychina in Alcoholism.**—The formula used by Prof. A. Flint, of the Bellevue Hospital, in chronic alcoholism is as follows:

℞ Nitrate strychnia........................grs. viii
Acid salicylic..............................grs. iv
Alcohol.......................................℥i
Water ........................................℥iii

M. Make up antisiptically (?). 15 minims of this mixture equals $\frac{1}{16}$ grs. strychnia. Sig. 15 minims hypodermically two or three times a day. "Strychnia," says the Columbus *Medical Journal*, "has become the drug *par excellence* for these cases, and its effect is so striking and so decided in cases of alcoholism, that it has become to be regarded as a specific antidote to alcohol poisoning. Its immediate effects, when given hypodermically, are most satisfactory and surprising. In the form and dose in which it is administered it will clear up nearly every case of alcoholism in from twenty-four to forty-eight hours."

**Asylum Changes.**—Superintendent B. M. Worsham has been transferred from the Southwest Texas Lunatic Asylum, at San Antonio, to the Austin asylum, and succeeds Dr. C. T. Simpson, resigned. Dr. W. W. McGregor, of Laredo, has been appointed superintendent of the Southwest Texas Lunatic Asylum at San Antonio. Dr. McGregor is a graduate of the Albany, New York, Medical College of the class of 1873. He is a physician of prominence in Southwest Texas, where he has been residing, but so far as we are aware, has never had any experience in the management of the insane.

---

**The Charbon.**—This dread plague is prevailing in certain portions of Louisiana, and is very fatal. Governor Culberson, of course, quarantined against it. In his proclamation he orders the carcasses of all animals dying of the disease in this State to be burned. This is a wise step, as the germ of the disease has been known to survive burial of the animals thirty years. In the trenches around Paris mules dead of charbon were buried, and it is said that thirty years afterward, upon the bones being unearthed, living animals became infested by the poison which had remained so long dormant, and the disease lighted up. Darwin has shown that earth worms bring these disease germs to the surface, and that outbreaks of charbon have been caused in that way.

---

GALVESTON, TEXAS, August 27, 1896.

*To Members of the Texas State Medical Association:*

I have been requested by Dr. C. A. L. Reed, of Cincinnati, O., upon authority of the International Executive Committee of the United States, to give notice that I will issue delegates' certificates to such member of our State Association as may apply to me for the same to the Second Pan-American Medical Congress, to be held in the City of Mexico, November 16 to 19, inclusive, 1896. With the understanding that I am authorized to do so, it will give me pleasure to furnish such credentials to all members in good standing who may wish to attend.

Fraternally yours,
H. A. WEST, Secretary,
2020 Market street.

---

**Pasteur Monument.**—Of special interest to physicians, scientists and Franco-Americans.

Galveston, August 22.—Announcement of the Pasteur Monument Committee of the United States: It has been decided to erect, in one of the squares of Paris, a monument to the memory of M. Pasteur. The Paris committee has wisely determined that the statue, obtained through international effort, shall be located at Paris, where it will be seen by the greatest number of his countrymen and also by his numerous admirers from other lands. The Paris committee has for honorary members the President of the Republic and his cabinet, together with about 160 of the most prominent officials, scientists, and other distinguished citizens of France. The Paris committee has extended the opportunity to the people of the United States to assist in this tribute of appreciation and love. The committee does not consider it necessary to urge any one to subscribe. The contributions of Pasteur to science and to the causes of humanity were so extraordinary and are so well known and appreciated in America, that they believe our people only need the opportunity in order to demonstrate their deep interest. All can unite in honoring Pasteur. He was such an enthusiastic investigator, so simple, so modest, so lovable, and yet so earnest, so great, so successful. His ideals were so high, and his efforts to ameliorate the condition of humanity were so untiring, that the committee anticipates an enthusiastic response from the whole civilized world. No one is expected to contribute an amount so large that it will detract in the least from the pleasure of giving. A large number of small subscriptions freely contributed, and showing the popular appreciation of this eminent Frenchman, is what the committee most desires. The undersigned has been appointed by Dr. Geo. M. Sternberg, Surgeon-General of the United States Army, as an associate member of the committee for Texas, and will take pleasure in receiving and forwarding subscriptions. The amounts thus far subscribed by individuals vary from 50 cents to $10. The original subscription lists will be forwarded to Paris for preservation.

H. A. West, M. D.,
2020 Market Street.

---

**A Rare Opportunity** is offered a competent and energetic physicians to secure one of the best locations to practice to be found anywhere. Dr. Sam R. Burroughs, who has held the practice of the larger part of east Leon county for twenty-five years, has been induced to go to the railroad (Buffalo) for convenience of practice, and offers, for less than cost of residence, his home, consisting of a well improved farm of 206 acres, with growing crops, seven-room residence nearly new, out houses, bath house, milk house, stables, three tenant houses, fine orchard, his office and equipments—all for $1500. His practice and good will go to the purchaser. Churches and schools; healthy home beautifully improved. Here is a rare chance to secure a lovely home and paying practice for a small consideration. Address Dr. Burroughs, at Buffalo, Leon county, Texas, or this journal.

---

**Von Boeckmann** authorizes us to say that the forthcoming Transactions being in paper, he will bind it, if desired, on individual orders, for 50 cents per volume. Every member is entitled to a copy, and if orders are sent in before the work is delivered to the Secretary, it will save express charges both ways.

